# Prevalence of depression and associated factors among elderly people in Womberma District, north-west, Ethiopia

**DOI:** 10.1186/s12888-021-03145-x

**Published:** 2021-03-08

**Authors:** Nebiyu Mulat, Hordofa Gutema, Gizachew Tadesse Wassie

**Affiliations:** 1 Womberma District Health Office, Dembecha, Amhara Region Ethiopia; 2 Department of Health promotion and Behavioral sciences, School of Public Health, College of Medicine and Health Sciences, Bahir Dar University, Bahir Dar, Ethiopia; 3 Department of Epidemiology and Biostatistics, School of Public Health, College of Medicine and Health sciences, Bahir Dar University, Bahir Dar, Ethiopia

**Keywords:** Prevalence, Depression, Elder, Geriatrics, Ethiopia

## Abstract

**Background:**

Depression is a common mental disorder that suffers many elderly people who are neglected, their problems are overlooked, and no efforts are made to mitigate their suffering. It is a mental health problem which is both underdiagnosed and under treated in primary care settings. This study was aimed to assess the prevalence and associated factors of depression among elderly people in Womberma District, Ethiopia.

**Methods:**

A community based cross-sectional study was conducted from March 10/2020 - April 08/2020. From a 2269 study population, 959 study participants were selected by using computer-generated simple random sampling techniques from selected *kebeles*. Data were collected using Geriatric depression scale item 15 through face-to-face interviews. Then, entered into EpiData version 3.1 and then exported to SPSS version 23.0 for analyses. Bi-variable and multivariable logistic regression models were fitted to identify associated factors of depression. An adjusted odds ratio with 95% confidence interval was reported and statistical significance was declared at *P*-values < 0.05.

**Results:**

The prevalence of depression among elderly people was 45% [95% CI: 41.7–48.5%]. Being female [AOR = 1.60, 95% CI [(1.15–2.23)], (age > =75 years [AOR = 7.95, 95% CI (4.98–12.68)], age 70–74 years [AOR = 5.52, 95% CI (3.52–8.66)], age 65–69 years [AOR = 2.39,95% CI (1.54–3.70)]; divorced [AOR = 2.53, 95% CI (1.59–4.03)], widowed [AOR = 2.65, 95% CI (1.61–4.34)]; poor social support [AOR = 3.32, 95% CI (1.77–6.23)] and presence of known chronic disease [AOR = 1.91, 95% CI (1.30–2.81)] were significantly associated factors with depression.

**Conclusions:**

In this study, the prevalence of depression among elderly people was high compared with previous studies done in other parts of Ethiopia. Older age, being female, marital loss, presence of known chronic disease, and poor social support were contributing factors for depression among elders. Early screening and co-morbidity management of depression should be comprised in basic primary health care packages. And also, ensuring adequate social support by establishing the Geriatrics care center could play a crucial role to mitigate the suffering of the elders from marital loss provoked loneness and depression.

## Background

Depression is a mental disorder characterized by feelings of depressed mood, loss of interest or pleasure in activities, and loss of energy at least for two weeks [1]. The elderly people with depression present with alteration in feeding or sleep, sense of worthlessness, or repeated thoughts of death or self-harm attempt [1]. Psychiatric problems are usually results from social, and occupational distresses [2].

Depression is one of the mental health problem in elderly people and it results in an increased risk of suicide [3]. The burden of depression in Nigeria and Egypt was 44.7 44.4% [4, 5] and in Ambo and Harar Ethiopia, it was 41.8 and 28.5% [6, 7] respectively.

In Ethiopia, the burden of depression among elder adults is not well addressed. This gap may contribute to poor or in- consistent mental health care at the community level [8].

Individuals with age older than sixty years in developing countries are considered as elderly [9] and also the world’s population is speedily aging, between 2015 and 2050, the number is projected to increase from 900 million to two billion [10].

Mental disorders among elderly adults contribute to 6.8% of the total disability and approximately 15% of them suffer mainly from depression [10]. The burden of depressive disorders among the elderly is estimated to range between 10 and 29% depending on different socio-cultural situations [1]. And it is projected to be the first cause of years lived with disability in 2020 [11].

In most sub-Saharan Africa countries, though families traditionally had been the primary care and support for elder people, increased mortality of working-age adults from the ongoing different infectious disease epidemic weakening the support network [12, 13].

Elderly adult faces many problems which include physical problems, psychological problems, nutritional problems like anemia and malnutrition, socio-economic problems [14]. These health problems lead to various disabilities found that about one-third of the elderly are suffering from psychiatric illnesses and depression alone accounts for more than 50% [15].

There is an increased burden of diseases affecting different systems with the advancement of age, apart from that economic loss, dependency on others, loss of self-worth perpetuate sufferings of elder age [16, 17]. Depression is one of them which amplifies functional disabilities [18]. An estimated 800,000 people died due to suicide every year, which is the worst complication of depression [19, 20].

In most developing countries, depression issues are neglected within health care policy, planning, and only limited resources are allocated to mental health services [21, 22]. And, it is both underdiagnosed and undertreated in primary care settings [3].

In Ethiopia, though psychiatry problem is the leading non-communicable disorder and national mental health policy has been launched, interventions against the problem are not yet significant [20, 23].

The studies on depression among elders undertaken in Ethiopia are not only a few but also narrow and limited in towns [6, 7]. This study aimed to assess the prevalence and associated factors of depression on individuals whose age is 60 years and above in both urban and rural communities of Womberma District.

## Methods and materials

### Study area

This study was conducted in the Womberma District which is found in the west Gojjam zone of the Amhara region, and located 427 km from Addis Ababa. The district has 21 kebeles (lower administrative unit in Ethiopia) of which 19 are rural and two are urban and there are 30,785 households in the district.

The total population of the District is 132,375, of which the rural population is 116,412 and the urban population is 15,963, (66,849 females and 65,526 males). And a total of 5457 are with the age of ≥60 years from community health information system/CHIS/ registration [24].

### Study design and period

A community based cross-sectional study was conducted from March 10/2020 - April 08/2020.

### Source population and study population

The source population for this study was all elderly people who live in Womberma District, whereas the study population was all elderly people live in the Womberma District of selected kebeles.

### Inclusion criteria and exclusion criteria

Elderly people age 60 years and above who live in the district and available during the study period were included. Elderly people who unable to communicate were excluded from the study.

### Sample size determination

The sample size of this study was determined by using EPI-INFO Version 7 software, considering the following assumptions: case to control ratio: 1:1, power: 80, % of outcome in the unexposed group, AOR, and 95% CI. Sex, marital status, occupation, and living arrangement were variables used from previous studies which were significantly associated with depression [6]. The variable sex gave the largest sample size, 436. Then, by adding design effect two and 10% non-response rate, the final sample size was 959 individuals.

### Sampling technique and procedure

A multistage sampling technique was used. First, the kebeles were classified as two urban and 19 rural. Then, 50% from urban and 40% of rural kebeles were selected by using a simple random sampling technique. Finally, by proportional allocation to each kebele, individuals were selected by computer generating simple random sampling.

### Study variables

#### Dependent variable

Depression (Yes = 1, No = 0).

#### Independent variables

Socio-demographic characteristics: Age, Sex, Ethnicity, Marital status, occupation, income, religion, Living arrangement, Educational status, Family size, and residence.

Clinical, Psychosocial, and substance use-related factors: chronic medical illness (hypertension, heart disease, diabetes mellitus, epilepsy, HIV/AIDS), social support, a good relationship with neighbors, Loneliness, history of mental illness, Consumption of psychoactive substances like alcohol drinking, cigarette smoking, and khat chewing; sleep medication, Physical disability.

#### Operational definitions

Elder age: Those participants who are older than or equal to 60 years old were considered as elder age people [10].

Depression: It was measured by 15 items of the geriatric depression scale (GDS) and operationalized as not depressed if they scored below five and depressed if they scored five and above. Scores of 0–4 were considered as normal, 5–8 mild; 9–11 moderate; and 12–15 severe depression [25, 26]. In the Geriatric depression scale, 15 items five of questions are negatively worded questions. So, if participants respond as yes, it is recorded as “0” and if no, it is coded as “1”. On the other hand, the remaining positive GDS item 15 questions were labeled as if yes “1” and if no, “0”.

Perceived social support: Social support has been described as support access to an individual through social ties to other individuals, groups, and the larger community. Perceived social support was operationalized as the following by using the Oslo-3 scale and individuals score, 3–8 as Poor social support, Moderate social support if they score 9–11, and Strong social support if they score 12–14 [27].

Substance use: Alcohol, tobacco, and khat use were measured using the WHO Alcohol, Smoking, and Substance Involvement Screening Test (ASSIST) (version 3.1) [28]. The ASSIST risk score ranges from 0 to 31 for tobacco smoking and 0 to 39 for alcohol drinking and khat chewing. The risk score of the respondents obtained for alcohol is categorized into ‘low’ (0 to 10), ‘moderate’ (11 to 26) or ‘high’ risk (27+); for tobacco products ‘low’ (0 to 3), ‘moderate’ (4 to 26) and ‘high’ risk (27+) and for khat ‘low’ (0 to 3), ‘moderate’ (4 to 26) and ‘high’ risk (27+) [28].

Wealth index: Is a composite measure of the cumulative living standard of a household. It was calculated using data on a household’s ownership of a set of assets, such as animals, television, radio, lands, televisions, bicycles, and cars [29].

### Data collection tools

Socio-demographic characteristics, wealth index, clinical, substance use, and psychosocial factors of elderly people were collected through face to face interviews. Alcohol, smoking, and substance involvement screening test was designed for using across different cultural settings. The instrument’s psychometric properties have been tested using data from multiple countries, including low, middle, and high-income countries, and shown to be valid, reliable, and easy to administer across settings [30]. Psychoactive substance use was considered if the participants used substances like alcohol, khat, cigarettes in the preceding last 3 months by using ASSIST [28].

We had also assessed clinical conditions that might contribute to depression such as hypertension, diabetes mellitus, heart diseases, epilepsy, and HIV/AIDS. Geriatric depression scale item 15 has been extensively tested and validated in low and middle-income countries such as India, Nepal, and other Asian countries [15, 31, 32]. It has been also checked in Ethiopian regions by previous studies [6, 7]. Geriatric depression scale item 15 was used to assess the presence of depression among elderly people who have good sensitivity and positive predictive values for the diagnosis of major depression consisting of 15 questions [33].

The data were collected from study participants by face to face interviews from house to house. The questionnaire was developed in English and translated into the local language (Amharic) by language translator and translated back to English to keep the consistency before the actual data collection. Data were collected by seven clinical nurses who currently work in health centers and was supervised by two public health officers.

### Data processing and analysis

The questionnaire was coded and entered into Epi-Data version 3.1 and exported to SPSS version 23 for further analysis [34]. Descriptive statistics, such as frequencies, prevalence, and measure of central tendency according to the nature of data were presented. A binary logistic regression technique was used. Variables with less than 0.20 *p*-values in the bivariable analysis were fitted to the multivariable logistic regression for final analysis. The adjusted odds ratio (AOR) with 95% CI was reported and statistical significance was considered at *P*-values < 0.05. A variable inflation factor (VIF) was used to check the presence of multicolinearity between independent variables at maximum threshold of 10 and no multicolinearity was detected. Model fitness was checked by using Hosmer and Lemeshow goodness of fit test (*p*-value was 0.394). Tables, graphs, and texts were used for data presentation.

### Data quality assurance

The questionnaire was pre-tested on 5% of the sample size in the adjacent Burie District to check the understandability of the questionnaires. One-day training was given to data collectors and supervisors on the study instrument, data collection procedure, and the ethical principles of confidentiality. Two more additional visits were made if a respondent would not found in the first visit then replaced by other respondents. The collected data were reviewed and checked for completeness before data entry.

## Results

### Socio-demographic characteristics

A total of 941 participants were involved with a response rate of 98.1%. Half of (50.8%) participants were females. The mean age of the participants was 69.04 (SD ± 6.602) years. More than half (55.5%) were married, 880(93.5%) were followers of Orthodox Christianity, 520 (55.3%) were living with their spouses, while 328 (34.8%) were living with their children. The majority, 860(91.4%) of the participants had no formal education. Most of the participants (85.6%) were rural residents. More than two-thirds (73%) of the respondents were farmers. Around one fifth (20.1%) of the respondents were in the richest category (Table 1).

**Table 1:** Socio-demographic characteristics of elderly people at Womberma District, North West, Ethiopia, 2020 (*n* = 941)

Characteristics	Categories	Frequency	%
Sex	Male	463	49.2
	Female	478	50.8
Age	60–64 years	256	27.2
	65–69 years	249	26.4
	70–74 years	225	24
	> = 75 years	211	22.4
Residence	Urban	135	14.4
	Rural	806	85.6
Religion	Orthodox	880	93.5
	Muslim	55	5.8
	Protestant	6	0.7
Educational status	No formal education	859	91.4
	Primary education	55	5.8
	Secondary and above	27	2.8
marital status	Married	523	55.5
	Single	12	1.3
	Divorced	181	19.2
	Widowed	225	24
Living arrangement	Spouse	520	55.3
	Children	328	34.8
	Alone	93	9.9
Occupational status	Farmer	692	73.5
	Retired	74	7.8
	Merchant	19	2
	Others	156	16.7
Wealth index	Poorest	188	19.9
	Poor	189	20
	Medium	186	19.7
	Rich	188	20.3
	Richest	190	20.1
Family size	One	78	8.3
	Two	195	20.8
	Three	212	22.6
	Four	227	24
	Five and above	229	24.3

### Clinical, perceived psychosocial support and substance use

**Fig. 1:**
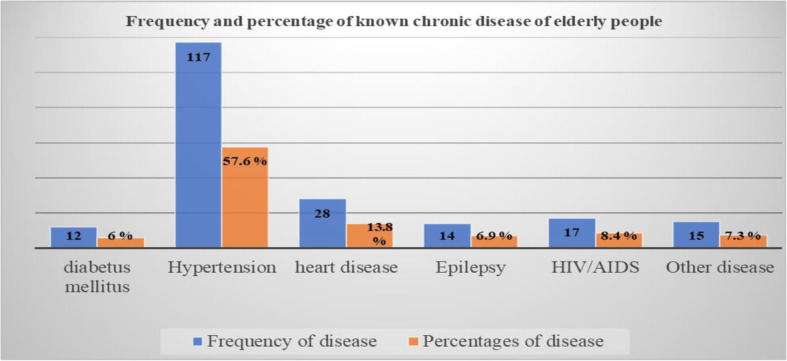
Frequency and percentage of known chronic disease by type of the illness among those elderly people with the previous history of chronic disease in Womberma District, North West, Ethiopia, 2020

The majority, 910(96.7%) of the respondents had no family history of mental illness. Two hundred three (21.6%) had a history of known chronic disease, of which 117 (57.6%) were hypertensive cases (Fig. 1). The majority, (98.4%) of the respondents didn’t use sleep medications. The majority, 838 (89%) of them had a good relationship with neighbors. Eight hundred thirty-five (88.7%) of respondents had ever used alcohol but 922(98%) of respondents had no ever used tobacco smoking. Nearly two-thirds of the participants (70%) had a moderate level of health risk of khat chewing. Half of the participants, (50%) had perceived poor social support, while (8.6%) had strong social support. All of the respondents didn’t use drugs by injection for non-medical use.

**Fig. 2:**
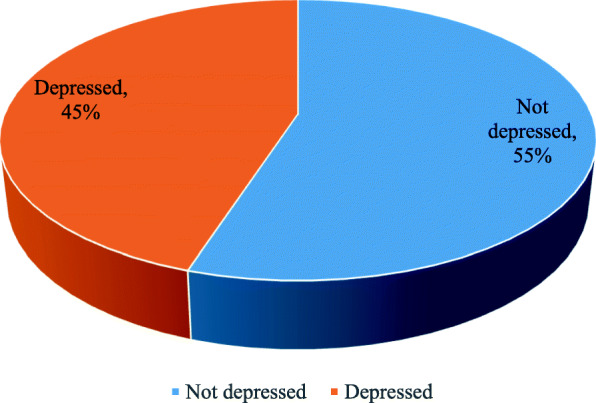
Percentages of depression among elderly people in Womberma District, North West Ethiopia, 2020

The prevalence of depression among elderly people was found to be 45% (95% CI: 41.7–48.5%) (Fig. 2). From depressed elderly people, 268(63%) were mildly depressed, 93(22%) were moderately depressed, while 63(15%) of them were severely depressed (Table 2). The prevalence of depression among urban elderly people was 45.1% and it was 45% among rural resident elders.

**Table 2:** Clinical, perceived social support and psychoactive substance use of elderly people at Womberma District, North West, Ethiopia, 2020 (*n* = 941)

Variable	Categories	Frequency	%
Family history mental illness	Yes	31	3.3
Known chronic disease	Yes	203	21.6
Sleep medication	Yes	15	1.6
Physical disability	Yes	23	2.4
A good relationship with neighbors	Yes	838	89
Feeling of loneliness	Yes	134	14
Perceived social support	poor social support	472	50
	Moderate social support	390	41.4
	Strong social support	79	8.6
Depression classification	Mild depression	268	63
	Moderate depression	93	22
	Severe depression	63	15
Ever used tobacco	Yes	19	2
Level of tobacco risk	Low	7	37
	Moderate	8	42
	High	4	21
Ever used alcohol	Yes	835	88.7
Level of alcohol risk	Low	483	57.8
	Moderate	334	40
	High	18	2.2
Ever used khat	Yes	50	5.3
Level of khat risk	Low	11	22
	Moderate	35	70
	High	4	8

### Factors associated with depression among the elders

In this study sex, age, marital status, having known chronic disease and poor social support were factors significantly associated with depression among the elders.

Female elders were 1.6 times more likely to develop depression compared to males (AOR: 1.60, 95% CI: 1.15–2.23). Elders whose age is greater than or equal to 75 years were eight times (AOR: 7.95, 95% CI: 4.98–12.68), 70–74 years were 5.52 times (AOR: 5.52, 95% CI: 3.52–8.66), and 65–69 years were 2.4 times (AOR: 2.39, 95% CI: 1.54–3.70) more likely to develop depression compared to 60–64 years old.

Divorced elders were 2.53 times (AOR = 2.53, 95% CI: 1.59–4.03), and widowed elders were 2.65 times (AOR: 2.65, 95% CI: 1.61–4.34) more likely to develop depression compared to married ones.

Elderly people who had known chronic disease were two times more likely to develop depression compared with their counterparts (AOR: 1.91, 95% CI: 1.30–2.81). And who had perceived poor social support were also three times more likely to had depression compared to those who had strong social support (AOR:3.32, 95% CI:1.77–6.23) (Table 3).

**Table 3:** Simple and multiple logistic regression analysis of depressive disorder among elderly people in Womberma District, North West, Ethiopia, 2020 (*n* = 941)

Variables	Categories	Depression		COR(95%CI)	AOR(95%CI)	*P*-value
		Yes	Yes			
Sex	Female	257	221	2.06 (1.58–2.67)	1.60 (1.15–2.23)	0.005*
	Male	167	296	1	1	
Age	> = 75 years	142	69	7.88 (5.19–11.96)	7.95 (4.98–12.68)	< 0.001**
	70–74 years	138	87	6.07 (4.05–9.10)	5.52 (3.52–8.66)	< 0.001**
	65–69 years	91	158	2.20 (1.48–3.28)	2.39 (1.54–3.70)	< 0.001**
	60–64 years	53	203	1	1	
Occupational status	Retired	41	33	1.67 (1.03–2.70)	0.70 (0.38–1.28)	0.247
	Merchant	6	13	0.62 (0.23–1.65)	0.41 (0.13–1.30)	0.131
	Others(/gov/t, NGO/daily labor/	82	74	1.49 (1.05–2.11)	0.62 (0.37–1.02)	0.064
	Farmer	295	397	1	1	
Marital status	Single	4	8	1.06 (0.31–3.59)	0.82 (0.16–4.19)	0.820
	Divorced	112	69	3.46 (2.43–4.91)	2.53 (1.59–4.03)	< 0.001**
	Widowed	141	84	3.57 (2.58–4.96)	2.65 (1.61–4.34)	< 0.001**
	Married	167	356	1	1	
Family size	Five and above	99	130	0.50 (0.29–0.84)	2.11 (0.47–9.38)	0.322
	Four	96	131	0.48 (0.28–0.81)	1.57 (0.35–6.91)	0.551
	Three	83	129	0.42 (0.25–0.72)	1.20 (0.27–5.38)	0.806
	Two	99	96	0.68 (0.39–1.15)	1.68 (0.37–7.52)	0.493
	One	47	31	1	1	
Living arrangement	Children	173	155	1.89 (1.42–2.50)	0.85 (0.56–1.30)	0.469
	Alone	58	35	2.80 (1.78–4.42)	1.30 (0.32–5.29)	0.712
	Spouse	193	327	1	1	
Known chronic disease	Yes	126	77	2.41 (1.75–3.32)	1.91 (1.30–2.81)	0.001*
	No	298	440	1	1	
Physical disability	Yes	14	9	1.92 (0.82–4.49)	1.86 (0.62–5.53)	0.263
	No	410	508	1	1	
Sleep medication	Yes	4	11	0.43 (0.13–1.38)	0.28 (0.06–1.17)	0.083
	No	420	506	1	1	
A good relationship with neighbors	No	56	47	1.52 (1.00–2.29)	1.17 (0.70–1.95)	0.540
	Yes	368	470	1	1	
Feeling of loneliness	Yes	72	62	1.50 (1.04–2.16)	1.00 (0.62–1.61)	0.972
	No	352	455	1	1	
Perceived social support	poor	276	196	4.15 (2.42–7.12)	3.32 (1.77–6.23)	< 0.001**
	Moderate	128	262	1.44 (0.83–2.49)	1.24 (0.66–2.34)	0.498
	Strong	20	59	1	1	
Ever used tobacco	Yes	6	13	0.55 (0.21–1.47)	0.39 (0.12–1.27)	0.120
	No	418	504	1	1	

* *P* value < 0.05, ** *p* value < 0.001, Hosmer and Lemeshow goodness of fit test (*p*-value = 0.394)

## Discussions

The prevalence of depression in this study was 45%, which was in line with studies done in rural Nigeria (44.7%) and Egypt (44.4%), [4, 5] respectively. But it was higher than studies done in Ambo (41.8%), Harar (28.5%) and China (10.5%) [6, 7, 35] respectively. This variation might be due to tool variation, as in China, the study used GDS 30 to screen depression and most of the participants were married and live with their spouses. Thus, from both studies, being married was found less prone to depression among the elders. In Ambo, it might be due to a difference in the study population; that is most of the participants were males because being male was less prone to depression compared to females in both findings.

Besides, this finding was higher than studies done in urban Sri Lanka among 60–74 years (13.9%) [36] and China (32.8%) [37]. This difference might be because in Sri Lanka; the study populations were 60–74 age. Individuals with age above 74 years were more likely to develop depression compared to having less age; not only in our finding but also in different works of the literatures [4, 38]. In china, the source of difference might be from classification variation to have depression that is they used 6 and above score to have depression using GDS 15 which in turn might cause an underestimation of depression prevalence.

But this finding was lower than results from India (52.5%) [39], Nepal (57.8%) [40], Urban India (75.5%) [41], Vietnam (66.9%) [42], Portuguese (61.4%), and Brazil (49.76%) [43]. These disparities might explain by different reasons like most of the participants in our finding were married than in Nepal and India. Because being married was less prone to depression compared to divorced and widowed in both studies. In urban India, it might be due to depression measurement tool variations and study population. In Portugal and Brazil, it might be due to the difference in study population that participants who had long stay in institutions and most of the participants were female sex and had no partner. Because being female sex and being divorced or widowed were more prone to have depression in both studies. In Vietnam, the difference might be due to tool variation as they used the Zung self-rating depression scale to screen depression [42].

Factors statistically associated with the outcome variable depression among the elders in this study were: Sex, age, marital status, having known chronic disease, and poor social support. Elderly females were more prone to have depression than males. This evidence agreed with the evidence revealed from Brazil, Sri Lanka, and India [32, 44, 45] respectively. The possible explanations for the discrepancies between females and males being exposed to depression because of biological differences, that is females affected by pregnancy and related physiological changes [32]. And also females take the majority of household responsibilities, and they depend on men economically; particularly in poorer settings [44].

Age was another significant predictor of depression which is in line with findings from Brazil, Vietnam, Sri Lanka, and India [36, 39, 42, 45] respectively. It is known that as people get older, they faced many problems which include physical, psychological, nutritional and socio-economic problems. These health problems lead to various disabilities found that about one-third of the elderly are suffering from psychiatric disorders [15]. Apart from that economic loss, dependency on others, loss of self-worth perpetuates the suffering of elder age [17].

Marital status of the elders was statistically significant predictor of depression. Both divorced and widowed elders were more prone for depression than the married ones. This finding was supported by studies in Sri Lanka and South Africa [36, 44]. This phenomenon might be attributed from the perceived loneliness sensation and loss of social support [36].

Elders who had known chronic diseases were at higher odds of developing depression compared with their counterparts. The result was in line with Fuzhou China and Sri Lanka [35, 36]. According to WHO, the presence of chronic illness is one of the risk factors for developing depression [46]. This could be attributed to the fact that physical illness could increase the development of emotional problems or depression.

Furthermore, we have found that poor social support was one of the contributing factors of depression among elder populations. This study was in line with studies from Sri Lanka and India [36, 39] respectively.

### Limitations of the study

Some of historical questions could be prone to recall bias. And, variables like wealth index, alcohol drink, Khat chewing, and cigarette smoking are a sensitive issues and might cause social desirability bias though we used separate room for interview.

## Conclusions

In this study, the prevalence of depression among elderly people was high compared with previous studies done in other parts of Ethiopia. Older age, being female, marital loss, presence of known chronic disease, and poor social support were contributing factors for depression among elders. Hence, depression is very common in elderly people with a chronic physical illness like hypertension, diabetes mellitus, and heart diseases, screening and co-morbidity management of depression should be comprised under basic primary health care packages. Besides ensuring adequate social support by establishing a Geriatrics care center could play a crucial role to mitigate the suffering of the elders from marital loss and poor social support provoked loneness and depression.

## Abbreviations

AOR: Adjusted Odds Ratio
ASSIST: Alcohol Smoking and Substance Involvement Screening Test
CES-D: Centre Epidemiological Studies Depression Scale
CI: Confidence Interval
COR: Crude Odds Ratio
DCR: Diagnostic Criteria
GDS: Geriatric Depression Scale
HIV/AIDS: Human Immune Virus /Acquired Immune Deficiency Syndrome
SPSS: Statistical Package for Social Science
USA: United States of America
WHO: World Health Organization
ZDSRS: Zung Depression Self-Rating Scale
