# Correction to: Prevalence of depression and associated factors among elderly people in Womberma District, north-west, Ethiopia

**DOI:** 10.1186/s12888-021-03205-2

**Published:** 2021-04-16

**Authors:** Nebiyu Mulat, Hordofa Gutema, Gizachew Tadesse Wassie

**Affiliations:** 1 Womberma District Health Office, Dembecha, Amhara Region Ethiopia; 2 Department of Health promotion and Behavioral sciences, School of Public Health, College of Medicine and Health Sciences, Bahir Dar University, Bahir Dar, Ethiopia; 3 Department of Epidemiology and Biostatistics, School of Public Health, College of Medicine and Health sciences, Bahir Dar University, Bahir Dar, Ethiopia

**Correction to: BMC Psychiatry 21, 136 (2021)**

**https://doi.org/10.1186/s12888-021-03145-x**

Following publication of the original article [1], the authors identified an error in Table 3. The correct table is given below.

**Table 3:** Simple and multiple logistic regression analysis of depressive disorder among elderly people in Womberma District, North West, Ethiopia, 2020 (*n* = 941).

Variables	Categories	Depression		COR(95%CI)	AOR(95%CI)	*P*-value
		Yes	No			
Sex	Female	257	221	2.06 (1.58-2.67)	1.60 (1.15-2.23)	0.005*
	Male	167	296	1	1	
Age	>= 75 years	142	69	7.88 (5.19-11.96)	7.95 (4.98-12.68)	<0.001**
	70-74 years	138	87	6.07 (4.05-9.10)	5.52 (3.52-8.66)	<0.001**
	65-69 years	91	158	2.20 (1.48-3.28)	2.39 (1.54-3.70)	<0.001**
	60-64 years	53	203	1	1	
Occupational status	Retired	41	33	1.67 (1.03-2.70)	0.70 (0.38-1.28)	0.247
	Merchant	6	13	0.62 (0.23-1.65)	0.41 (0.13-1.30)	0.131
	Others(/gov/t, NGO/daily labor/	82	74	1.49 (1.05-2.11)	0.62 (0.37-1.02)	0.064
	Farmer	295	397	1	1	
marital status	Single	4	8	1.06 (0.31-3.59)	0.82 (0.16-4.19)	0.820
	Divorced	112	69	3.46 (2.43-4.91)	2.53 (1.59-4.03)	<0.001**
	Widowed	141	84	3.57 (2.58-4.96)	2.65 (1.61-4.34)	<0.001**
	Married	167	356	1	1	
Family size	Five and above	99	130	0.50 (0.29-0.84)	2.11 (0.47-9.38)	0.322
	Four	96	131	0.48 (0.28-0.81)	1.57 (0.35-6.91)	0.551
	Three	83	129	0.42 (0.25-0.72)	1.20 (0.27-5.38)	0.806
	Two	99	96	0.68 (0.39-1.15)	1.68 (0.37-7.52)	0.493
	One	47	31	1	1	
Living arrangement	Children	173	155	1.89 (1.42-2.50)	0.85 (0.56-1.30)	0.469
	Alone	58	35	2.80 (1.78-4.42)	1.30 (0.32-5.29)	0.712
	Spouse	193	327	1	1	
Known chronic disease	Yes	126	77	2.41 (1.75-3.32)	1.91 (1.30-2.81)	0.001*
	No	298	440	1	1	
Physical disability	Yes	14	9	1.92 (0.82-4.49)	1.86 (0.62-5.53)	0.263
	No	410	508	1	1	
Sleep medication	Yes	4	11	0.43 (0.13-1.38)	0.28 (0.06-1.17)	0.083
	No	420	506	1	1	
A good relationship with neighbors	No	56	47	1.52 (1.00-2.29)	1.17 (0.70-1.95)	0.540
	Yes	368	470	1	1	
Feeling of loneliness	Yes	72	62	1.50 (1.04-2.16)	1.00 (0.62-1.61)	0.972
	No	352	455	1	1	
Perceived social support	poor	276	196	4.15 (2.42-7.12)	3.32 (1.77-6.23)	<0.001**
	Moderate	128	262	1.44 (0.83-2.49)	1.24 (0.66-2.34)	0.498
	Strong	20	59	1	1	
Ever used tobacco	Yes	6	13	0.55 (0.21- 1.47)	0.39 (0.12-1.27)	0.120
	No	418	504	1	1	

* *p* value <0.05, ** *p* value <0.001, Hosmer and Lemeshow goodness of fit test (*p*-value = 0.394)

The author group has been updated above and the original article [1] has been corrected.
